# Epidemiology of Classic and Novel Human Astrovirus: Gastroenteritis and Beyond

**DOI:** 10.3390/v9020033

**Published:** 2017-02-18

**Authors:** Diem-Lan Vu, Albert Bosch, Rosa M. Pintó, Susana Guix

**Affiliations:** 1 Enteric Virus Laboratory, Department of Genetics, Microbiology and Statistics, University of Barcelona, Barcelona 08028, Spain; Diem-Lan.Vu@hcuge.ch (D.-L.V.); abosch@ub.edu (A.B.); rpinto@ub.edu (R.M.P.); 2 Nutrition and Food Safety Research Institute (INSA-UB), University of Barcelona, Santa Coloma de Gramenet 08921, Spain

**Keywords:** astrovirus, zoonosis, gastroenteritis, encephalitis, meningitis, epidemiology

## Abstract

Since they were identified in 1975, human astroviruses have been considered one of the most important agents of viral acute gastroenteritis in children. However, highly divergent astroviruses infecting humans have been recently discovered and associated with extra-intestinal infections. The report of cases of fatal meningitis and encephalitis, especially in immunocompromised individuals, has broadened their disease spectrum. Although zoonotic transmission among animal and human astroviruses has not been clearly recognized, the genetic similarity between some human and animal viruses makes it likely to occur. This review provides an update on the epidemiology of both classic and novel human astroviruses, and a comprehensive view on confirmed or potential association between astrovirus and human disease.

## 1. Introduction

Since their first discovery in 1975 in the stool samples of children with diarrhea [1], human astroviruses (HAstVs) have been well-established etiological agents of viral gastroenteritis with a worldwide distribution [2,3]. They are small, non-enveloped, single-stranded positive RNA viruses and they make up the *Astroviridae* family. To date, the family has been divided into two genera: *Mamastrovirus* and *Avastrovirus*, including viruses infecting mammals and birds, respectively. Their genome codes for three open reading frames (ORFs), with ORF1a and ORF1b encoding the nonstructural protease and polymerase proteins, respectively, and ORF2 encoding the capsid proteins. While they have been reported in cases of adult gastroenteritis, they are predominantly considered a common cause of viral gastroenteritis in the pediatric population, after rotavirus and norovirus. In addition to children, HAstV gastroenteritis also frequently occurs in the elderly [4] and in immunocompromised individuals [5,6,7,8].

Since 2008, two novel groups of highly divergent astroviruses, named MLB (Melbourne) and VA/HMO (Virginia/Human-Mink-Ovine-like), have been identified in human stool of individuals with diarrhea using next-generation sequencing (NGS) [9,10,11,12,13,14]. Although these new viruses were initially isolated in children with gastroenteritis, the number of systematic epidemiological studies to determine their true prevalence is still low, and no definitive association between novel astroviruses and gastroenteritis has yet been established. In addition, both classic, but especially novel, HAstVs have recently been identified as the cause of unexpected central nervous system (CNS) infections in vulnerable individuals, highlighting that these viruses may bypass the gastrointestinal tract and infect other tissues and organs [15]. 

The aim of this review is to describe the recent findings on both classic and novel HAstV in terms of viral diversity, tropism, disease association, and prevalence, and identify potential knowledge gaps for future investigations. 

## 2. Classification 

The level of amino acid identity between MLB-AstVs, VA/HMO-AstVs, and classic HAstVs is very low (Table 1), suggesting that there may be significant biological and antigenic differences between them. On average, identities between MLB and classic HAstVs are 33%, 54%, and 27% for ORF1a, ORF1b, and ORF2, respectively. For VA, the average identities with classic HAstVs are slightly lower (24%, 52%, and 24% for each ORF, respectively). 

Classical HAstVs are classified into eight serotypes (HAstV-1 to HAstV-8) with 64%–84% capsid amino acid similarities between them. According to the International Committee on Taxonomy of Viruses (ICTV), this group defines *Mamastrovirus 1* species within the *Mamastrovirus* genus (Table 1). Although serotype-specific neutralizing antibodies may be detected in sera from infected individuals, suggesting a lack of heterotypic immunity, further studies are required to assess whether cross-protection may occur between serotypes. Within each serotype, different genetic lineages or subtypes can also be identified, based on a lower than 93%–95% nucleotide homology of partial ORF2. Lineage classification has been recently reviewed in [2,16], with six lineages within HAstV-1 (1a to 1f), four within HAstV-2 (2a to 2d), two within HAstV-3 (3a and 3b), three within HAstV-4 (4a to 4c), three within HAstV-5 (5a to 5c), and two within HAstV-6 (6a and 6b). The subsequently-identified HAstV-3c should be added to the classification [17]. Whether there is a significant biological difference between lineages is still poorly understood; although not fully addressed yet, some of them may share some *ORF1a1b* genes and recombination may significantly contribute to enhanced diversification and evolution. A classification system based on both ORF1b and ORF2, similar to what has been established for similar viruses, such as noroviruses [18], would be extremely informative.

Compared to classic HAstVs, novel HAstVs are even more diverse. MLB-HAstVs (*Mamastrovirus 6*) is classified in three types or clades (MLB1, MLB2, and MLB3), while VA HAstVs are divided in *Mamastrovirus 8* species, containing VA2 (also named HMO-B) and VA4, and *Mamastrovirus 9* species containing VA1 (also named HMO-C) and VA3 (HMO-A) [2,15,19]. Although not yet officially recognized by the ICTV, and based on the capsid homology, the recently identified VA5 clade [20,21] may be classified as a new species. Since no specific antisera against novel HAstVs are available, the correlation between these clades and serotypes has not yet been experimentally confirmed. 

## 3. Diversity and Zoonotic Potential 

**Figure 1:**
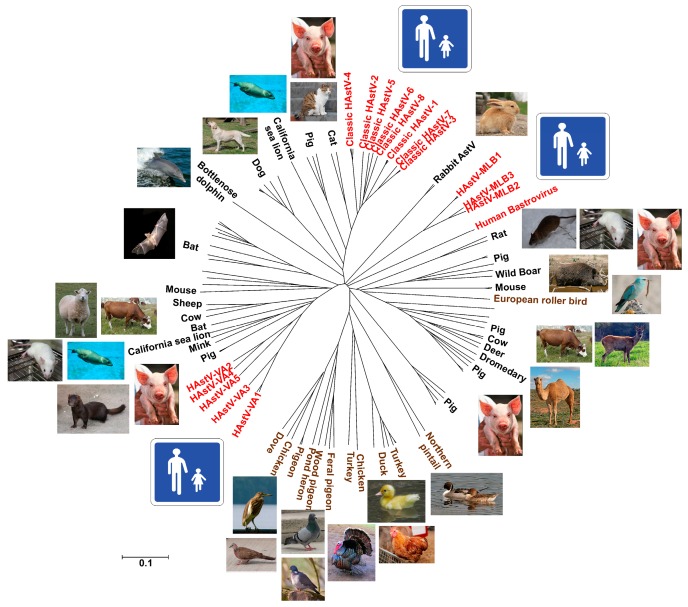
Phylogenetic tree of representative members of the *Astroviridae* family. The tree was constructed based on complete capsid amino acid sequences, using the Neighbor Joining method implemented in the MEGA6 program [27]. The tree is drawn to scale, with branch lengths in the same units as those of the evolutionary distances (p-distance) used to infer the phylogenetic tree. All positions containing alignment gaps and missing data were removed only in pairwise sequence comparisons (pairwise deletion option). Viruses infecting birds are shown in brown, while viruses infecting humans are shown in red.

Astrovirus demonstrate a high genetic diversity leading to the potential infection of a large spectrum of mammals and birds [22]. Figure 1 provides an exhaustive view of astrovirus host species and reveals that the distance between distinct virus species infecting humans is higher than the distance between HAstVs and some of those infecting animal species. Bats and pigs harbor the highest astrovirus strain diversity, suggesting their role as potential reservoirs. Astrovirus infecting bats are not known to infect other species, but a recombinant virus between pig and HAstVs have been described, suggesting that they may have crossed the species barrier [23]. Emerging diagnostic tools, such as NGS, continue to discover additional astrovirus host species [24,25,26]. It remains unknown if these findings reflect astrovirus genetic evolution, or if these viruses were formerly unrecognized because they were not targeted by the detection assays.

The high genetic variability of HAstVs, together with the occurrence of recombination events during concurrent infections with multiple strains, makes them serious candidates for emerging zoonotic infections. Cross-species transmissions are especially frequent in avian viruses [28]. A recent investigation on non-human primates shows that they can be infected by different astrovirus strains closely related to those usually infecting either other mammals, avian species, or even humans, including the novel HAstV MLB [29]. In humans, antibodies directed toward non-human astroviruses have been identified [30]. Thus, there is certainly an overlap between animal and human astrovirus strains, and there are more and more data suggesting that astrovirus infections are not species specific. Interestingly, a novel astrovirus-like virus has recently been detected in stools of individuals infected with the human immunodeficiency virus (HIV) and healthy individuals [31]. This new virus, tentatively named Bastrovirus, shows the closest homology to HAstV MLB at the capsid level, and to members of the *Hepeviridae* family at the nonstructural proteins level, pointing to a putative recombination event between members of these two close viral families.

## 4. Pathogenesis and Disease Spectrum

### 4.1. Astrovirus in the Gastrointestinal Tract

Classic HAstVs are a frequent cause of mild gastroenteritis in children under two to five years of age, usually causing a self-limiting disease, notably two- to three-day watery diarrhea. Human and turkey astrovirus capsid protein have been shown to act like an enterotoxin and induce intestinal epithelial barrier dysfunction, according to in vitro and animal models, respectively [32,33]. Vomiting is less prevalent in astrovirus infection than in rotavirus or norovirus infection, and the incubation period is a little bit longer. In neonates, an association between HAstV infection and necrotizing enterocolitis has been observed by two independent studies [34,35]. 

The causal link between novel HAstVs and acute gastroenteritis has been studied in case-control studies, with inconsistent results. While Holtz et al. did not find any association between MLB1 and diarrhea in a cohort of Indian children [36], Meyers et al. reported an association for MLB1, but not for MLB2, VA1, and VA2 in Kenya and Gambia, and even a higher prevalence of MLB3 in control subjects [20]. While Holtz et al. did not observe differences in viral load between cases and controls, the reported titers (7 × 10^3^ and 4 × 10^4^ RNA copies/mL of fecal suspension) were strikingly lower than those usually found in classic HAstVs infections. Whether HAstV MLB1 replicates to a lesser extent than classic HAstVs, and if this contributes to the lack of association with diarrhea, if any, remain to be clarified.

In addition, the fact that astrovirus can be recovered in feces of asymptomatic children [20,37,38,39] and mammals [40] raises the question of whether it simply reflects astrovirus prolonged shedding or nucleic acid persistence, or if astrovirus virions can persist in some way in the gastrointestinal tract, and be part of the gut virome. If so, the determinants of such a persistent infection need to be explored, as well as those that can trigger an increase in viral replication or a recurrent pathogenic infection, as this can potentially lead to severe local or disseminated infection [41]. Further studies confirming the qualitative or quantitative role of novel HAstVs in gastroenteritis are imperative.

Finally, the role of astrovirus as part of the gut virome, interacting with other component of the gut microbiome and the immune system should also be taken into account for further studies. In the poult enteritic syndrome [42], turkeys demonstrate growth retardation and behavioral changes that potentially derive from digestive disorders, but could also be linked to the gut-brain axis [43]. Yet, astrovirus is frequently identified in co-infection with other enteric pathogens [20,44], with potentially complex pathogenesis involving transkingdom interactions [45], as suggested by Qureshi et al. in their study demonstrating a reduced uptake of *Escherichia coli* by macrophages in presence of turkey astrovirus [46]. 

### 4.2. Astrovirus beyond the Gastrointestinal Tract and in Other Organs

Beside the gastroenteritis classically seen in children and the elderly, HAstVs have recently been associated with encephalitis and meningitis in immunocompromised patients (reviewed in Reference [15]); this is particularly true for the VA1/HMO-C genotype, which has been identified in five cases of encephalitis so far [47,48,49,50,51], but also for the MLB group [52,53] and the classic HAstVs [41]. Table 2 summarizes these nine cases of CNS infection in humans. Only one case affected an immunocompetent adult woman, who recovered from a MLB2 self-limited CNS infection. Among the remaining cases, six of them were fatal. Close contact with animals and young children, intravenous immunoglobulin treatment and the stem cell graft have all been suggested as possible source of infection, but none has been confirmed. Viral load in brain biopsy specimen can be as high as 1.5 × 10^7^ RNA molecules per reaction [47], sometimes 10^3^-fold higher than in cerebrospinal fluid (CSF) and 10^6^-fold higher than in stool [48]. These results point out that analysis of superficial samples, such as CSF or stools, can be insufficient and that analysis of profound specimens (brain biopsies) may be required in order to make a diagnosis.

In animals, astrovirus has also been associated with CNS infection, causing suppurative encephalitis in several bovid [54], the shaking-mink syndrome [55], and potentially being associated with congenital tremor syndrome in piglets [56]. Thus, astrovirus’ tropism for the CNS appears to be highly plausible. Like enteroviruses, HAstVs may cause a wide disease spectrum, depending on specific virus and host factors, which remain to be characterized and which may ultimately determine the outcome of infection.

Virus–host interaction studies disclosing how adaptive and innate immunity control HAstV infection and dissemination beyond the gastrointestinal tract are in progress. Adaptive immunity has been shown to be important both in human and animal studies [57,58,59]. In mice deficient in adaptive immunity, viral replication in the gastrointestinal tract is increased, and viral RNA has also been detected in the mesenteric lymph nodes, spleen, liver, and kidney. No RNA has been identified in extra-digestive organs of wild-type mice [58]. The importance of innate immune responses (type I interferon ) in limiting viral replication has been recently shown by in vitro studies for classic HAstVs [60] and in vivo for avian and mice viruses [61,62].

Finally, NGS analysis has also allowed the identification of MLB2 and VA1 in plasma or nasopharyngeal swabs of children with fever and acute respiratory disease of unknown etiology [63,64,65]. Again, large prospective prevalence investigations are needed to better understand the clinical relevance of these findings.

Given the wider tissue tropism and disease spectrum of animal astroviruses (for a review see References [66,67]) together with their high zoonotic potential, close attention should be paid to the emergence of strains that could cause unexpected diseases in humans.

## 5. Prevalence and Distribution

### 5.1. Classic HAstVs

Classic HAstVs are ubiquitous but their burden is noteworthy in developing countries. Prevalence among children with gastroenteritis, in studies using real time PCR (RT-PCR) screening method, is about 5% (ranges from 0 to over 20%) (Table 3). Although HAstV incidence seems to decrease over the last decades, higher positivity rates are still observed in areas such as China or South America [68,69]. Prevalence is higher among outpatients compared to hospitalized children. HAstV is estimated to contribute to nosocomial infections in approximately 5% of cases [70,71]. For unknown reasons, studies using multiplex RT-PCR for screening tend to generate lower prevalence [72,73,74,75].

Although controversial, HAstV infection mostly occurs during winter season. Epidemiological studies have shown a higher burden of HAstV diarrhea every other year. Regarding the incidence of different serotypes, HAstV-1 is by far the most prevalent type worldwide, accounting for over 50% of cases in all recent reports (Table 3), while the second most frequent differs depending on geographical localizations. Geographical and temporal distribution of the different genetic lineages within each serotype, including HAstV-1, has not been systematically addressed: Whether some lineages are constantly prevalent or whether a turnover of lineages occurs over time is unclear. While some HAstV-1 strains have been detected for 10 consecutive years in the same area [101], some other lineages have been shown to emerge or re-emerge over time [102]. Systematic lineages surveillance would help to understand the driving forces of HAstV evolution. 

With the drastic increase in sensitivity of real-time RT-PCR method, a significant higher positivity rate of HAstVs among asymptomatic children has been reported [88,90,103], being approximately 4%, but reaching up to over 20% in some studies [38] (Table 3). Accurate viral load quantification will be important to determine whether the degree of viral replication may contribute to explain the clinical outcome of HAstV infection. In this regard, a community-based birth cohort study performed in Tanzania, using TaqMan array cards targeting 19 enteropathogens, showed no association between diarrhea and qualitative results, but found a significant association for astrovirus, rotavirus and *Shigella*/enteroinvasive *E. coli* with quantitative results [104]. As already mentioned, co-infections of HAstVs with other enteric viruses are often reported, and there are data indicating a significant correlation between co-infections and clinical disease [90]. Of note, the average rate of HAstV co-infections, especially with enteric viruses, is over 30%, making association with gastroenteritis more challenging. 

### 5.2. Major Reported Outbreaks

Although classic HAstVs are typically involved in sporadic cases of gastroenteritis, and although most viral gastroenteritis outbreaks worldwide are caused by noroviruses [105], noteworthy HAstV outbreaks have also been described worldwide. Yet, HAstV has been implicated in 0.5% of all acute gastroenteritis outbreaks reported from 1994 to 2005 in the Netherlands [106]. A prospective study in the US also shows that HAstVs may cause 10% of all reported outbreaks in day-care centers [107]. Outbreaks classically occur in schools, nursing homes and hospitals [4,106,108,109,110], but can also affect neonatal care units and maternity wards [111,112]. Interestingly, an outbreak among young healthy adults in a riot police camp in Korea was caused by HAstV serotype 5, which is rarely identified in sporadic cases of HAstV gastroenteritis [113]. 

### 5.3. Novel Astroviruses

Novel HAstVs have also been identified worldwide, without significant differences between industrialized and developing countries. A recent review on novel astrovirus epidemiology [15] shows an overall positivity rate in stool lower than those observed for classic HAstVs (1.5%) (Table 4). In only one study from Japan, MLB-HAstVs have been found in a higher proportion than classic HAstVs (10.6%) [84]. Unexpectedly, despite this apparent low overall prevalence, serological studies in the US have reported a surprisingly high HAstV-MLB1 and HAstV-VA1 (HMO-C) seroprevalence (86% and 65%, respectively) [114,115]. Thus, the development and optimization of sensitive diagnostic methods and the more systematic screening for novel HAstVs are mandated to provide a valuable estimate of their prevalence.

The publication of several case reports of meningitis and encephalitis caused by novel HAstVs infection has prompted research groups to retrospectively screen largely for novel HAstVs in CSF samples [48,52]. The observed positivity rate is low (0–0.2%), suggesting, that either HAstVs CNS infection is of very rare occurrence, or the targeted population and/or type of specimen are not adequate. 

## 6. Transmission Routes

Classic HAstVs are essentially transmitted through the fecal–oral route, as demonstrated by human volunteer studies [119,120]. HAstV has also been proven stable enough in the environment to undergo vehicular transmission through drinking [121] of fresh or marine water [122]. They can be found in sewage [123,124] and, like other viral pathogens, are not completely removed from sewage after wastewater treatment [125]. Prevost and co-workers reported median viral loads of 2.69 × 10^3^ genome copies/L, similar to other common enteric viruses, such as noroviruses, in wastewater treatment plant effluents in Paris [126]. Novel HAstVs have also been commonly detected in sewage samples, although their prevalence may be lower than classic HAstV [127,128], reflecting either a lower number of infected individuals, a lower viral shedding and/or persistence, or less sensitive detecting methods. In any case, both classic and novel HAstV are ultimately discharged into fresh and marine water environments [129,130,131]. 

Surface and ground waters are routinely employed as source of drinking water, representing a major public health threat when water quality is poor. According to the World Health Organization (WHO), children in developing area are particularly vulnerable to gastroenteritis attributable to unsafe water, and poor sanitation and hygiene [132]. As such, endemic waterborne astrovirus represents a significant cause of digestive illness as demonstrated by a French surveillance study [133]. Infectious HAstVs have also been detected in tap water in Ghana [134] and in dam water where sewage effluents from an area reporting concurrent gastroenteritis outbreak circulate [131]. A report from Slovenia described HAstVs in 1.4% of drinking groundwater supplies and in 27% of surface water samples [135].

The public health risks associated with bathing and other recreational activities in sewage-polluted waters is much lower than for consumption of contaminated water, but is, nevertheless, not negligible. A study performed in the UK revealed a much higher HAstV seroprevalence in surfers than in the control population [136]. In addition, investigation of an outbreak of gastroenteritis in children and adults bathing in an outdoor pool in Helsinki revealed water contaminated with HAstVs and noroviruses [137]. 

Infrequently, HAstVs have been implicated in large foodborne gastroenteritis outbreaks. One report describes as many as 4700 individuals infected by contaminated food from a common supplier [109]. Similar to other foodborne viruses, the matrices most often associated with HAstV outbreaks are live bivalve mollusks, grown and harvested in polluted environments, salad greens and soft fruits irrigated with contaminated waters. 

Inadequate food handling leads to contamination of ready-to-eat food products that are consumed with little, if any, cooking process. HAstV infections may result in asymptomatic virus shedding in adults too [138,139,140], and asymptomatic food handlers are more frequently the source of foodborne outbreaks than symptomatic ones, whatever the causing virus is [141]. 

In institutional settings such as hospitals, day-care centers or geriatric centers, fomites play an important role on the vehicular transmission of HAstV [142,143], which may persist dried on inanimate surfaces long enough and at sufficiently high viral load to represent a significant health threat [144] to vulnerable hosts. Classic HAstVs may survive for at least two days at room temperature in non-porous materials, such as toilet tiles, and for at least one week in porous materials, such as toilet paper or bed linen [144].

## 7. Control and Prevention

Prevention of HAstV infections mostly relies on the control of virus transmission. The best measure to avoid person-to-person transmission is the frequent hand washing with soap and water, especially after using the toilet and changing diapers, and before eating or preparing food. Disinfection of potentially contaminated fomites is also highly recommended. Alcohol (90%) has been proven to be useful for non-porous fomites and hand disinfection [145]. Unfortunately, to date, there are no data on survival and inactivation methods of novel HAstVs, but the use of bleach is recommended. 

Astrovirus detection and inactivation in water and food are also the best means to prevent waterborne and foodborne astrovirus outbreaks. Although several methods for the detection and quantification of HAstVs exist, they are not routinely screened in at-risk water and food matrices. Classic HAstV demonstrate a prolonged survival in drinking water and disinfection with 1 mg/mL of free chlorine for two hours is required for successful inactivation [121]. Of note, the environmental persistence differs between strains, Serotypes 6 and 7 (genogroup B based on ORF1a phylogeny), have an increased resistance to chlorine disinfection compared to serotypes 1–5 and 8 (genogroup A based on ORF1a phylogeny) [124]. 

To prevent disease development, vaccination and potentiation of the natural defenses, such as the gut microbiota, are thought to be the most efficient measures. However, there are no available commercial vaccines for HAstVs. Virus-like particles (VLP) for some classic strains, produced in different recombinant expression systems have been described [146,147], which could be good antigen candidates for an inactivated vaccine. Recently, two subunit vaccine candidates, based on the spike P protein of an avian astrovirus fused together with the spike P protein of hepatitis E virus and the spike P protein of a norovirus or the VP8 protein of rotavirus, have been described to elicit a good IgG response in mice [148,149]; a similar approach could be intended with HAstV. Yet, the lack of commercial interest in HAstV vaccine production may rely on the low clinical impact of the classic HAstV infections in healthy patients and on the need of a multivalent vaccine to cover all circulating serotypes or at least the most prevalent. As for the novel HAstVs, although they may be clinically more relevant, their low prevalence does not draw the attention of the vaccine manufacturing industry.

Treatment for serious gastroenteritis consists of oral or intravenous fluid replacement to avoid dehydration. Immunocompromised hosts with severe or persistent diarrhea may receive intravenous immunoglobulins, although their true efficacy has yet to be established in large-scale studies [150]. To date, there is no known specific treatment for novel astrovirus CNS infection; corticosteroids, ribavirin, and PEG-interferon have been described in case reports [49,50] without a clear efficacy. Consequently, the current measures include supportive treatment and restoration of immunity. The emergence of neuroinvasive astrovirus strains requires investigations for the successful treatment of severe CNS complications. 

Water treatments with chlorine and synthetic flavonoids have been proven to impair the multiplication of human astroviruses [121,151]; the effect of natural extracts from the plant *Achyrocline bogotensis* [152] on astrovirus replication has also been demonstrated, but none of these compounds have been tested in vivo. 

## 8. Conclusions 

HAstVs should no longer be considered as only a virus causing mild gastroenteritis in children. Reports of life-threatening CNS infections associated with HAstVs, together with the large panel of host species, their potential broad disease spectrum, and high transmissibility reinforce the hypothesis that astroviruses may emerge and evolve to cause unrecognized diseases in humans. The development of sensitive diagnostic methods, such as broadly reactive reverse transcription quantitative PCR (RTqPCR) assays, of immunological reagents and cell culture-adapted strains should be the next priorities to better understand their evolution and pathogenesis.

On the one hand, HAstVs should still be epidemiologically monitored with special attention to inter-species transmission. Since diagnostic interpretation has gained complexity due to the higher sensitivity of RTqPCR assays, which have been firmly implemented in routine diagnostics, additional case-control studies would be valuable to evaluate whether there is a correlation between viral load and symptoms and assess the role of asymptomatically infected individuals as a virus reservoir. Disease correlation and quantitative analyses should also be performed in case of mixed infections. On the other hand, HAstVs should also be monitored in immunocompromised patients with CNS infection of unknown etiology: definitive diagnosis is crucial to better estimate the burden of HAstV in unexplained infectious encephalitis, and to identify potential modifiable risk factors that could be important for prevention. 

## Figures and Tables

**Table 1:** Amino acid sequence identity between classic HAstVs, MLB-HAstVs (Human astrovirus Melbourne) and VA-HAstV (Human astrovirus Virginia), for the 3 open reading frames (ORFs). Representative members of each group were used for calculations (Classic: L23513, L13745, AF141381, AY720891, DQ028633, HM237363, Y08632, AF260508; MLB: FJ222451, JF742759, JX857870; VA2-VA4: GQ502193, JX857869; VA1-VA3: FJ973620, JX857868; VA5: KJ656124. The grouping of serotypes and clades into the different International Committee on Taxonomy of Viruses (ICTV) recognized species within the *Mamastrovirus* genus is indicated.

*Mamastrovirus* Species	Classic	MLB	VA2-VA4	VA1-VA3	VA5
	*Mamastrovirus 1*	*Mamastrovirus 6*	*Mamastrovirus 8*	*Mamastrovirus 9*	Unassigned
Serotypes/Clades	HAstV-1 to 8	MLB1, MLB2 and MLB3	VA2 (HMO-A) and VA4	VA1 (HMO-C) and VA3 (HMO-B)	VA5
**ORF1a (protease and other nonstructural proteins)**					
Classic	100	–	–	–	–
MLB	32.8	100	–	–	–
VA2-VA4	24.1	29.1	100	–	–
VA1-VA3	24.2	28.9	67.4	100	–
VA5	23.9	28.2	61.5	59.6	100
**ORF1b (RNA dependent RNA polymerase)**					
Classic	100	–	–	–	–
MLB	54.5	100	–	–	–
VA2-VA4	51.8	49.4	100	–	–
VA1-VA3	53.0	49.3	73.7	100	–
VA5	50.2	50.7	74.0	71.5	100
**ORF2 (capsid proteins)**					
Classic	100	–	–	–	–
MLB	27.5	100	–	–	–
VA2-VA4	24.0	21.9	100	–	–
VA1-VA3	23.0	22.1	51.9	100	–
VA5	23.8	20.6	58.9	53.1	100

**Table 2:** Summary of the 9 case reports of HAstV infections causing severe central nervous system (CNS) infections in humans (adapted from [50] and updated).

Type of Novel HAstV	Year	Country	Age of Patient	Underlying Condition	Type of CNS Infection/Presentation	Treatment	IS/Other	Outcome	Reference
***Mamastrovirus 1***									
HAstV-4	2008	Switzerland	3 months	HSCT for severe combined immunodeficiency	Meningoencephalitis	None	Not described	Dead	[41]
***Mamastrovirus 6***									
MLB1	2015	Japan	4 years	CB HSCT for congenital aplastic anemia GvH disease	Encephalitis	Aciclovir IVIG Edaravone	Ciclosporin MMF	Alive	[53]
MLB2	2014	Switzerland	21 years	Healthy	Acute meningitis	Ceftriaxone Aciclovir	None	Alive	[52]
MLB2	2014	Switzerland	37 years	HSCT for acute myeloid leukemia, relapse	Meningitis	None	IT chemotherapy 5-AZC Cranial irradiation	Dead	[52]
***Mamastrovirus 9***									
VA1 (HAstV-PS)	2007	US	15 years	X-linked agammaglobulinemia	Headache, suicidal and homicidal ideation, memory loss, ataxia, progressive cognitive decline	None	Related to underlying disease	Dead	[47]
VA1	2013	UK	42 years	HSCT for chronic lymphocytic leukemia	Progressive sensorineural deafness Encephalitis	Valaciclovir BS antibiotics Steroids IVIG Ribavirin	Not described	Dead	[49]
VA1	2014	France	14 years	X-linked agammaglobulinemia	Four-year history of progressive cognitive impairment, ataxia and seizure.	IVIG Steroid Ribavirin PEG IFN alpha-2b	Related to underlying disease	Alive	[50]
VA1	2015	UK	18 months	HSCT for cartilage hair hypoplasia GvH disease	Encephalitis	Cidofovir * Adenovirus-specific DLI *	Ciclosporin MMF Steroids	Dead	[48]
VA1	2015	UK	8 months	HSCT for acute myeloid leukemia GvH grade 1	Encephalitis	DLI	Ciclosporin **	Dead	[51]

CB: Cord blood; HSCT: Hematopoietic stem cell transplantation; GvH: Graft-versus-host disease; IS: Immune suppression; MMF: Mycophenolate mofetil; 5-AZC: 5-azacitidine; BS: Broad spectrum; CNS: Central nervous system; CSF: Cerebrospinal fluid; HSCT: Human stem cell transplant; IT: Intrathecal; HAstV-PS: Human astrovirus Puget Sound; BMT: Bone marrow transplant; NP: Not performed; IVIG: Intravenous immunoglobulin; DLI: Donor lymphocytes infusion; * for adenovirus infection prior to CNS complication; ** tapered off at the time of CNS complication.

**Table 3:** Major epidemiological studies to determine the classic HAstV positivity rate among different populations, published over the last 10 years.

Geographical Area (Time of Study)	Type of Individuals	Method	Positivity Rate (%)	% of Positive Samples Containing Other Pathogens (Type)	Serotype Prevalences	Reference
**Children with symptoms of AGE**						
***Asia***						
China (2007–2008)	Outpatients < 15	RT-PCR	13.6	N/A	HAstV-1 (100%)	[76]
China (2008–2009)	Hospitalized < 5	RT-PCR	4.6	26 (other enteric viruses)	HAstV-1 (100%)	[77]
China (2010–2011)	Outpatients < 5	RT-PCR	1.8	50 (other enteric viruses)	HAstV-1 (100%)	[78]
China (2010–2011)	Outpatients < 5	RT-PCR	2.9	64 (rotavirus)	HAstV-1 (100%)	[68]
China (2005–2006)	<5	RTqPCR	9.1	N/A	HAstV-1 (96%); HAstv-3 (4%)	[79]
India (2004–2008)	Hospitalized < 5	RT-PCR	3.1	8.8 (rotavirus)	HAstV-1 (68%); HAstv-2 (10%); HAstV-8 (16%); HAstV-5 (6%)	[80]
Japan (2009–2013)	Outpatients < 5	RT-PCR	2.4	0	N/A	[81]
Japan (2009/10, 2014/15)	<15	RT-PCR	4.2	N/A	HAstV-1 (54%); HAstv-4 (23%); HAstV-8 (16%); HAstV-6 (7%)	[82]
Japan (2008/09, 2013/14)	Hospitalized < 15 with suspected viral gastroenteritis	RT-PCR	1.6	N/A	HAstV-1 (81%); HAstV-8 (16%); HAstV-3 (3%)	[83]
Japan (2012–2013)	Outpatients	RT-PCR	5.2	29 (other enteric viruses)	HAstV-1 (76%); HAstv-4 (24%)	[84]
Taiwan (2009–2011)	Hospitalized < 5	RT-PCR	2.6	20 (other enteric viruses)	N/A	[85]
Thailand (2000–2003, 2005, 2007–2008, 2010–2011)	Hospitalized < 5	RT-PCR	1.4	14 (rotavirus)	HAstV-1 (58%); HAstv-3 (21%); HAstV-5 (14%); HAstV-3 (7%)	[86]
Vietnam (2002–2003)	Hospitalized < 9	Multiplex RT-PCR	0.6	33 (other enteric viruses)	HAstV-1 (100%)	[72]
Vietnam (2005–2006)	Hospitalized and outpatients < 15	RT-PCR	13.9	28 (other enteric viruses)	HAstV-1 (100%)	[87]
***Africa***						
Burkina Faso (November 2011–September 2012)	Outpatients < 5	RTqPCR	4.9	7.7 (other enteric viruses)	HAstV-1 (42%); HAstv-2 (25%); HAstV-8 (25%); HAstV-5 (8%)	[88] *
Gabon (2010–2011)	Outpatients < 5	RT-PCR	6.3	55 (other enteric viruses)	HAstV-1 (89%); HAstv-4 (11%)	[89]
Ghana (November 2005–January 2006)	Outpatients < 5	RT-PCR	4.8	N/A	N/A	[90] *
Kenya and Gambia (2008–2009)	< 5	RT-PCR	2.7	N/A	N/A	[20] *
***Europe and Middle East***						
Bulgaria (summer 2009)	Hospitalized < 3, summer months	RT-PCR	6.9	50 (other enteric viruses, bacteria and parasites)	HAstV-1 (86%); HAstv-3 (14%)	[91]
Finland (2009–2010)	Children < 2 enrolled in prospective cohort INDIS Study	RTqPCR	1.9	33 (other enteric viruses)	N/A	[92]
Italy (2008–2009)	Hospitalized < 13	RT-PCR	2.1	0 (other enteric viruses)	HAstV-1 (73%); HAstv-2 (20%); HAstV-4 (7%)	[93]
Italy (2008–2009)	Hospitalized < 18	Multiplex RT-PCR	0	0	N/A	[73]
Moldova and Ukraine (2009)	Hospitalized < 5, negative for rotavirus	RTqPCR	1.4	14.3 (other enteric viruses)	HAstV-1 (80%); HAstv-8 (20%)	[94]
Qatar (June-November 2009)	Outpatients < 20	Multiplex RTqPCR	0.7	N/A	N/A	[74]
United Kingdom (2006–2007)	Hospitalized < 16, health-care associated AGE	RT-PCR	5	57 (other enteric viruses)	N/A	[71]
***Central and South America***						
Brazil (1994–1996; 1995–1999)	Outpatients < 6	RT-PCR	7.6; 29.7	22; 50 children with AGE and controls (other enteric viruses)	HAstV-1 (58%); HAstV-2 (24%); HAstV-8 (12%); HAstV-3 (6%)	[38] *
Brazil (1997–1999)	Outpatients < 2	RT-PCR	11	55 (other enteric viruses)	HAstV-1 (92%); HAstV-2 (2%); HAstV-3 (2%); HAstV-4 (2%); HAstV-5 (2%)	[95] *
Brazil (1994–1996; 1998–2002)	Hospitalized < 5	RT-PCR	4.3	30.4 children with AGE and controls (other enteric viruses)	N/A	[96] *
Brazil (2005–2011)	Children < 5, negative for rotavirus and norovirus	RT-PCR	7.1	N/A	HAstV-1 (70%); HAstV-2 (12%); HAstV-3 (10%); HAstV-8 (4%); HAstV-4 (2%); HAstV-6 (2%)	[69]
Venezuela (2003)	Outpatients < 5	Multiplex RT-PCR	1.5	29 (other enteric viruses)	HAstV-1 (67%); HAstV-3 (33%)	[75]
***North America***						
US (2006–2009)	Hospitalized and outpatients	RT-PCR	3.1	N/A	N/A	[8]
US (2008–2009)	Hospitalized and outpatients < 5	RTqPCR	4.9	25 children with AGE and controls (other enteric viruses)	HAstV-1 (52%); HAstV-2 (19%); HAstV-4 (8%); HAstV-8 (3%)	[39] *
**Children without diarrhea disorders**						
Burkina Faso (November 2011–September 2012)	Matched controls < 5	RTqPCR	2	N/A	HAstV-1 (42%); HAstv-2 (25%); HAstV-8 (25%); HAstV-5 (8%)	[88] *
Brazil (1997–1999)	< 2	RT-PCR	3	20 (other enteric viruses)	HAstV-1 (92%); HAstV-2 (2%); HAstV-3 (2%); HAstV-4 (2%); HAstV-5 (2%)	[95] *
Brazil (1994–1996; 1995–1999)	< 6	RT-PCR	20.7; 16.3	22; 50 children with AGE and controls (other enteric viruses)	HAstV-1 (58%); HAstV-2 (24%); HAstV-8 (12%); HAstV-3 (6%)	[38] *
Brazil (1994–1996; 1998–2002)	< 5	RT-PCR	0.5	30.4 children with AGE and controls (other enteric viruses)	N/A	[96] *
Ghana (November 2005–January 2006)	Matched controls < 5	RT-PCR	1.6	N/A	N/A	[90] *
Kenya and Gambia (2008–2009)	< 5	RT-PCR	2.4	N/A	N/A	[20] *
US (2008–2009)	Matched controls < 5	RTqPCR	3.0	25 children with AGE and controls (other enteric viruses)	HAstV-3 (57%)	[39] *
**Adults with AGE**						
China (2005–2006)	Collected from CDC’s surveillance	RTqPCR	5.4	N/A	HAstV-1 (96%); HAstv-3 (4%)	[79]
China (2007–2008)	Visiting an outpatient clinic and/or emergency room	RT-PCR	1.8	30 (other enteric viruses)	N/A	[97]
France (2010–2011)	Consulting a general practitioner	RT-PCR	6.9	50 (other enteric viruses)	N/A	[98]
Russia (2005–2007)	Hospitalized	RT-PCR	2.2	N/A	N/A	[99]
Singapore (October 2013–January 2014)	Hospitalized	RT-PCR	2	N/A	N/A	[100]
US (2006–2009)	Hospitalized and outpatients	RT-PCR	1.2	N/A	N/A	[8]
**Immunocompromised**						
Brazil (2003–2004)	HIV-seropositive children with and without diarrhea	RT-PCR	0; 11	0	N/A	[7]
US (2006–2009)	Hospitalized	RT-PCR	7.4	N/A	N/A	[8]

AGE: Acute Gastroenteritis; RT-PCR: Reverse Transcription Polymerase Chain Reaction; RTqPCR: Reverse Transcription Quantitative Polymerase Chain Reaction; N/A: Not available. * Case-control study.

**Table 4:** Major epidemiological studies to determine the MLB-HAstV and VA-HAstV positivity rate in stool samples.

Geographical Area (Time of Study)	Type of Individuals	Method	MLB Positivity Rate (%)	VA Positivity Rate (%)	Reference
**Children and adults with AGE**					
India (2005–2006)	Community-based samples from a birth cohort	RT-PCR	2.1	0.7	[12]
China (2004–2005)	Hospitalized patients	RT-PCR	0.2	0	[116]
China (2010–2011)	Outpatients < 5	RT-PCR	1.2	0.3	[68]
Japan (2012–2013)	Outpatient children	RT-PCR	10.6	0.6	[84]
Nepal (2006–2008)	Adults, negative for bacteria, rotavirus, adenovirus, HAstV, Giardia, Cryptosporidium or norovirus.	RT-PCR	0	2.1	[14] *
Kenya and Gambia (2008–2009)	Children < 5 from rural areas	RT-PCR	4.3	1.3	[20] *
UK (2013–2014)	Immunosuppressed and immunocompetent children and adults	RTqPCR (VA1)	N/A	0.3	[48]
Egypt (2006–2007)	Outpatients < 5	RT-PCR	1.4	0.5	[117]
Turkey (2004–2005)	Children < 5, negative for rotavirus	RT-PCR	0.7	0	[118]
Brazil (2005–2011)	Children < 2, negative for rotavirus and norovirus	RT-PCR	1	0	[69]
US (2008)	< 5	RT-PCR	0.6	0	[13]
**Children and adults without diarrhea disorders**					
Nepal (2006–2008)	Adults, negative for bacteria, rotavirus, adenovirus, HAstV, Giardia, Cryptosporidium or norovirus	RT-PCR	0	1	[14] *
Kenya and Gambia (2008–2009)	Children < 5 from rural areas	RT-PCR	6.4	1.8	[20] *
**Children with non-polio acute flaccid paralysis (AFP)**					
Nigeria (2006–2008)	Children < 15	RT-PCR	4.2	3.2	[14]
Pakistan (2006–2008)	Children < 15	RT-PCR	0	4.6	[14]
**Children and adults (undefined clinical presentation)**					
Switzerland (2014–2015)	Children and adults stool specimens stored at a Laboratory of Virology of a University hospital	RTqPCR (MLB2)	0.9	N/A	[52]

AGE: Acute Gastroenteritis; RT-PCR: Reverse Transcription Polymerase Chain Reaction; RTqPCR: Reverse Transcription Quantitative Polymerase Chain Reaction; N/A: Not available. * Case-control study.
